# Evaluating the Correlation between Overjet and Skeletal Parameters Using DVT

**DOI:** 10.1155/2012/921942

**Published:** 2012-07-31

**Authors:** Luca Lombardo, Chiara Sgarbanti, Antonio Guarneri, Giuseppe Siciliani

**Affiliations:** Postgraduate School of Orthodontics, University of Ferrara, Via Montebello 31, 44100 Ferrara, Italy

## Abstract

*Aim*. To evaluate the degree of correlation between a dental parameter of immediate clinical relevance (overjet) with skeletal (ANB angle) and dentoskeletal parameters such as the IMPA angle and upper incisor-bispinal angle. *Materials and Methods*. A sample of 42 subjects, all in complete permanent dentition and without a history of orthodontic treatment or systemic pathologies, was subdivided into 2 groups: group 1 consisted of 25 subjects with ANB angle 0°–4° (skeletal class I), and group 2 was made up of 17 subjects with ANB angle >4° (skeletal class II). Each subject underwent cone-beam computed tomography (CBCT). For each right and left CBCT, the following parameters were measured: (1) ANB, (2) OJ (overjet), (3) IMPA angle, and (4) upper incisor-bispinal angle (U1/ANS-PNS). *Results*. Analysis of the entire sample revealed that both right and left overjets were correlated in a statistically significant fashion (*P* < 0.001) with ANB. No correlation between overjet and IMPA emerged, while a weak correlation between overjet and the left U1-bispinal plane was ascertained. *Conclusions*. Overjet may be a reliable predictor of ANB, and to a lesser extent the U1-bispinal plane, particularly in skeletal class II.

## 1. Introduction

In order to formulate a correct orthodontic diagnosis, accurate and thorough documentation of anamnesis, intra- and extraoral clinical examinations, model analysis, radiographic analysis, and cephalometric and photographic studies is required [1].

In diagnosis, it is particularly important to obtain an accurate measurement of *overjet*, as this describes the sagittal relationship between the upper and lower central incisors. After crowding, excessive protrusion of the upper central incisors is the most frequent cause of malocclusion in Caucasian subjects [2]. It is generally accepted that increased overjet is due to a growth deficit of the jaw rather than poor positioning of the dental elements but no significant data regarding this has been published as yet [3].

*ANB* angle on the other hand indicates a skeletal discrepancy between the jaws, which must be brought back into harmony during treatment. This value, as well as being influenced by the anteroposterior relationship between the jaws, is not always an indicator of a real imbalance in the patient as it may also be influenced by the vertical height of the face or the position of the nasion [4]. However, consideration of another parameter, the inclination of the upper and lower incisors with respect to the osseous base, may help to establish the correct relative protrusion of the teeth [5].

Thus, a relationship between overjet and skeletal parameters is evident, but the challenge in planning adequate treatment can be understanding how to interpret and correctly correlate dental and skeletal values.

In this study, the following parameters were considered: overjet as the dental parameter, ANB as the skeletal parameter, and inclination of the incisors with respect to the osseous base, that is, the *IMPA* angle and the *U1-bispinal plane*  (*ANS/PNS*) angle, as the dentoskeletal parameters.

Values corresponding to these parameters were obtained from analysing of digital volumetric tomographs acquired via CBCT (cone-beam computed tomography). This system allows us to procure images suitable for detailed study of a patient's anatomy and to examine the structures present at a high level of accuracy and precision [6].

The aim of this study was therefore to evaluate the degree to which a dental parameter of immediate clinical relevance (overjet) correlates to skeletal (ANB) and dentoskeletal (IMPA angle and U1-bispinal plane angle) parameters to provide a potential diagnostic aid.

## 2. Materials and Methods

From a sample of 73 subjects aged between 18 and 40 years, *42* subjects (25 females, 17 males) all in integral permanent dentition, unaffected by maxillofacial syndromes or evident trauma, lacking a history of orthodontic or surgical treatment, and without insalubrious habits were selected; furthermore, all subjects with metallic prostheses in the incisal region were excluded, as these devices are known to provoke scattering phenomena, as were subjects with ANB angle <0°, as these subjects were not statistically significant in number. The sample was further divided into 2 groups: *group 1* comprising 25 subjects in skeletal class I with ANB angle 0°–4°, and *group 2* constituted by 17 subjects in skeletal class II, with ANB angle *x* > 4°.

CBCT (cone-beam computed tomography) was performed on each subject using the NewTom 3G Volume Scanner (QRsr1, Verona), which employs a conical beam X-ray emission technique that markedly reduces the quantity of radiation absorbed by the patient, to obtain the images. The settings applied were FOV 12 inches, 110 kV (AP-LL), 2.00 mA (AP), 1.00 mA (LL), exposure time 5.4 s, and section thickness 0.50 mm.

Each CBCT (cone-beam computed tomography) was then analysed via NNT NewTom 3G software by an orthodontist with experience in using this programme.

For each left and right CBCT (cone-beam computed tomography) the following parameters were measured:

- ANB angle: anterior-posterior relationship of the maxilla with the mandible is measured in degrees,
- overjet (OJ): sagittal relationship between the upper and lower central incisors is measured in millimeter,
- IMPA angle: inclination of the lower incisor with respect to the mandibular plane defined as axis between gonion (Go) and menton (Me) is measured in degrees,
- U1-bispinal plane angle: inclination of the upper incisor with respect to the bispinal plane. The bispinal plane is defined as axis between the anterior nasal spine (ANS) and the posterior nasal spine (PNS) is measured in degrees.

All CBCT (cone-beam computed tomography) measurements were subsequently repeated by the same operator in order to permit reliable evaluation of the data found. Moreover, to quantify the degree of error, an MSA (Measurement Systems Analysis) was performed which utilises a Dahlberg's *d* test, whose formula is *s* ^2^ = ∑*d* ^2^/2*n*.

Dahlberg's test revealed the absence of statistically significant systematic errors in measurement. However, the systematic error was subsequently calculated using Student's *t*-test for paired data and yielded a significance level of 0.05 (Table 1).

To measure the ANB angle, a secondary reconstruction was performed for each CBCT wherein 3D MIP (3D maximum intensity projection) images were created; this format was selected as it consents application of units of measurement to the image to be exported.

To measure overjet and IMPA, right and left panoramic sections in which the relationship between the upper and lower incisors was clear were taken, permitting identification of the menton and right and left Go for calculation of the IMPA and consenting the degree of overjet to be established.

**Figure 1:**
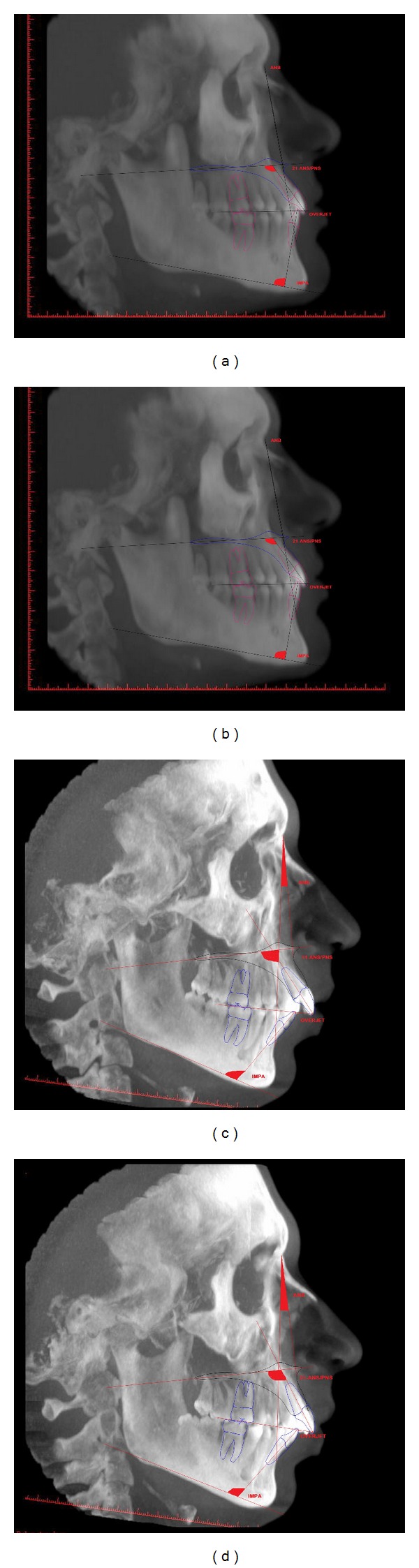
Examples of CBCT right and left of two subjects. The first two figures belong to a subject with ANB 0°–4° (skeletal class I), the second two to a subject with ANB >4° (skeletal class II). In the images, axes and angles like ANB, IMPA, U1-bispinal plane, and overjet were drawn to explain the aim of this work. In the research, each parameter was analysed via NNT NewTom 3G software using particularly secondary reconstruction.

Finally, in order to measure the inclination of the right and left upper central incisors with respect to the bispinal plane, a secondary reconstruction of each CBCT (cone-beam computed tomography) was obtained so as to procure sagittal sections of the upper jaw perpendicular to the line passing through the centre of each radicular canal. For each of these sections, the angle between the incisor axis and the bispinal plane, the latter passing through the anterior and the posterior nasal spines, was calculated (Figure 1).

## 3. Statistical Analysis

The data obtained (see Table 2) were subsequently analysed statistically using multiple linear regression in order to evaluate the existence of a statistically significant correlation between dental and skeletal parameters, both in the sample as a whole and for the disparate groups 1 (ANB angle: 0°–4°) and 2 (ANB angle > 4°).

Two distinct analyses were performed: the first to analyse the entire sample and the second to evaluate separately group 1 (ANB angle: 0°–4° mm) and group 2 (ANB angle > 4°). In both analyses, first OJ right then OJ left were assumed as dependent variables.

## 4. Results

### 4.1. Analysis of the Entire Sample

*OJ right* was equated to ANB, IMPA right, 11/ANS-PNS, and 21/ANS-PNS.

In *a distribution analysis*, only the dependent variable OJ right was found to have an altered trend. However, given the numerosity higher than 30, it was possible to hypothesise that the distribution of OJ right also tends towards the normal.

At this point, multiple linear regression analysis was carried out: first the *correlation indices* were applied and OJ right was found to correlate positively, to a statistically significant degree, with ANB (*R* = 0.4872; *P* = 0.001); then a multiple linear regression test revealed that OJ right only correlated in a statistically significant manner with ANB and 21/ANS-PNS (Table 3). Eliminating the two variables found to be nonsignificant, the following expression was formulated for the final model:

(1) OJ  right=−10.56+0.60∗ANB+0.10∗21/ANS-PNS,

with ANB (*P* = 0.000060) and 21/ANS-PNS (*P* = 0.003187) being highly significant, as was the intercept (*P* = 0.007069). Fisher's goodness-of-fit index also yielded a highly significant value (*P* = 0.00006). Nevertheless, the linear determination index was fairly low (*R* ^2^ = 0.391).

Subsequently *OJ left* was considered as the dependent variable and equated with ANB, IMPA left, 11/ANS-PNS, and 21/ANS-PNS. Via distribution analysis, the only variable found to have abnormal distribution was the dependent OJ left itself. However, given the numerosity higher than 30, it was possible to hypothesise that the distribution of OJ left also tends towards the normal.

Multiple linear regression analysis was also carried out for this variable: the correlation indices revealed that OJ left had a positive, statistically significant correlation with ANB (*R* = 0.4872; *P* = 0.001), and the multiple linear regression test showed that OJ left is correlated in a statistically significant manner with ANB and 21/ANS-PNS (Table 4). Elimination of the variable found to be nonsignificant (IMPA LEFT) yielded a final model which can be expressed as

(2) OJ  LEFT=−10.17+0.56∗ANB+0.10∗21/ANS-PNS,

with ANB (*P* = 0.000596), 21/ANS-PNS (*P* = 0.009593), and the intercept (*P* = 0.002) being highly significant. A highly significant value was also obtained upon application of Fisher's goodness-of-fit test (*P* = 0.0007), although the linear determination index was low (*R* ^2^ = 0.313).

### 4.2. Analysis of the 2 Groups

As per the entire sample, a linear regression model was employed for each group. 

In *group 1* (ANB  0 < *x* < 4), neither of the linear *correlation indices* OJ right and OJ left were found to be correlated to any of the predictor variables. Furthermore, *multiple linear regression analysis* for OJ right (Table 5) and OJ left (Table 6) showed that no independent variable was significant and, therefore, no model could be constructed.

In *group 2* (ANB > 4), analysis of the *correlation indices* yielded similar results. In fact, neither OJ right nor OJ left were found to correlate significantly with any of the predictor variables, although *multiple linear regression analysis* applied to OJ right revealed a statistically significant correlation between this parameter and ANB (Table 7). However, after elimination of the nonsignificant variables, regression analysis showed that no model could be constructed.

Linear regression analysis of OJ left, on the other hand, gave statistically significant values for ANB and 21/ANS-PNS (Table 8). Thus, eliminating the two nonsignificant values, a definitive final model was obtained in which ANB (*P* = 0.002990) and 21/ANS-PNS (*P* = 0.003550) were highly significant, as was the intercept (*P* = 0.003348). The model can therefore be expressed as

(3) OJ  LEFT=−22.03+1.05∗ANB+0.19∗21/ANS-PNS.

The indicator of linear determination *R* ^2^ is high enough (*R* ^2^ = 0.589) to validate the above model.

## 5. Discussion

Among the factors which must be evaluated to formulate a correct diagnosis and a suitable treatment plan, the anteroposterior relationship between the jaws is a particularly relevant parameter [7].

As proposed by the ABO (American Board of Orthodontist), overjet, in association with other parameters such as overbite, IMPA, presence of open bite or crossbite, or entity of crowding, is a useful indicator in evaluation of the diagnostic complexity [8].

The aim of this paper was to establish by what degree a dental parameter (overjet) is able to predict the entity of the skeletal parameter ANB and the dentoskeletal parameters IMPA and upper incisor-bispinal plane axis.

The sample in this study was constituted by subjects of the Dental Clinic of the University of Ferrara Postgraduate School in Orthodontics, all in integral permanent dentition, not treated orthodontically and not affected by systemic pathologies.

Subjects presenting skeletal class III were excluded as they were not present in a statistically significant number. This reflects the frequency of malocclusions present in the Italian population [9].

The use of CBCT (cone-beam computed tomography) was further justified by the fact that the effective dose of radiation that the patient receives (56.2 milli Sv) is significantly lower than that emitted by traditional fan beam systems (CT multislice 429.7 milli Sv) and is similar to values discharged during orthopantomography or conventional teleradiography (10.4 milli Sv) [10, 11].

From analysis of the entire sample, it emerges that overjet, both left and right, was correlated in a statistically significant manner (*P* < 0.001) with ANB, despite the index of linear determination being rather low: in the first case (right side) *R* ^2^ = 0.391, and in the second (left side) *R* ^2^ = 0.313. This was probably due to the fact that overjet is influenced by the inclination of the upper and lower incisors, while ANB also varies according to the anteroposterior position of the nasion [12], the inclination of the SN plane, and the inclination of the jaws [13]. Another factor able to modify the width of ANB, even if the relationship between the jaws remains constant, is the inclination of the occlusal plane [14].

In contrast, no correlation was revealed between overjet and IMPA, while a weak correlation emerged between overjet and left upper incisor-bispinal plane. This is likely to be due to the difficulty in establishing the position of the incisors with respect to the osseous base with a sufficient degree of reliability, as the dentoalveolar values are subject to greater compensatory variations [15, 16].

From the analysis of the two distinct groups, different results were obtained. Regarding skeletal first class subjects, it was not possible to formulate any type of correlation, neither on the left nor the right, between overjet and the other variables examined. This can be explained by the fact that skeletal class I subjects are generally less “harmonic,” that is, exposed to a greater number of compensatory variations, in both sagittal and vertical directions, sufficient to render the creation of a mathematical model able to predict their entity impossible [17–19].

Concerning skeletal class II on the other hand, it was possible to construct, for the left side, a good model to correlate overjet and ANB, characterised by a high significance value (*P* = 0.002990) and a good linear determination index *R* ^2^ = 0.589. Analogous to that revealed for the entire sample, no correlation with the IMPA and only a slight correlation with the left upper incisor-bispinal plane was determined.

In the study conducted by Zupancic et al. in exclusive class II subjects, the overjet was found to be “a statistically significant predictor of the skeletal relationship in the sagittal plane” [19]. However, in our study, it was not possible to construct a statistically significant model for the right-hand side. The difference between the two sides has no precise justification but is probably linked to the low number of test subjects in the sample.

In contrast to previous studies performed on conventional teleradiographs, the present study offers the advantage of analysing precisely and accurately the anatomical structures both from the left side and from the right, thereby permitting radiographic artefacts, superimpositions, and flaws produced by an inevitable and omnipresent asymmetry of the face to be eliminated [20, 21].

## 6. Conclusions

The results of the study conducted on the entire sample indicate how a dental parameter such as overjet can be a reliable predictor of ANB angle, and to a lesser extent the U1-bispinal plane. No significant data were revealed concerning IMPA.

However, if the cases of skeletal class I and class II are considered separately, different results are obtained. In fact, in the cases of skeletal class I (Group 1), it was not possible to establish a correlation between the variables analysed. In contrast, the class II cases reflected the entire sample in that OJ left was found to predict both ANB angle and 21/ANS-PNS to a sufficient degree, despite not being in correlation with IMPA.

## Figures and Tables

**Table 1:** Dahlberg's *d* test: *s* ^2^=∑*d* ^2^/2*n* applied to the entire sample and repeated twice revealed no statistically significant systematic measurement errors. The systematic error calculated via Student's* t*-test for paired data yielded a significance level of 0.05.

Variable	Dahlberg's test	*P* value
ANB	0.11905	0.950475
IMPA-r	0.53631	0.369756
11/SNA-SNP	0.64845	0.165493
OJ-r	0.00690	0.065285
IMPA-l	1.49048	0.673221
OJ-l	0.00417	0.617962
21/SNA-SNP	1.35000	0.098852

**Table 2:** Initial measurements of the entire sample revealed. In bold ANB >4, in regular font ANB 0–4.

Subject	ANB	IMPA-r	11/SNA-SNP	OJ-r	IMPA-l	OJ-l	21/SNA-SNP
**1**	**7.9**	**93.5**	**95.6**	**2.3**	**96.2**	**2.6**	**96.4**
2	1.8	86	97.6	0.8	81.5	0.8	96.5
3	2	85.6	93.7	2.1	82.5	2.1	100.1
4	1.7	92.2	105.7	1.7	97.8	1.3	112.9
**5**	**4.8**	**94.5**	**103.1**	**2.1**	**94.2**	**1.7**	**102.9**
**6**	**5.8**	**99.8**	**103.4**	**1.3**	**98.8**	**1.7**	**100.6**
**7**	**7.5**	**91.7**	**101.5**	**1.7**	**91.3**	**1.7**	**93.2**
**8**	**4.9**	**96.5**	**106.7**	**2.1**	**96.6**	**2.1**	**106.9**
9	2.5	94.3	110.9	2.6	94.4	2.1	110.6
10	3.3	98.8	100.3	3.5	99.5	3.5	104
11	3.8	94.6	100	1.7	98.8	1.7	100.5
12	1.5	87.8	100	0.8	90.5	0.8	95.3
**13**	**4.5**	**98.8**	**106.5**	**2.6**	**94.8**	**2.6**	**100.7**
**14**	**5**	**92.7**	**108.4**	**4.2**	**91**	**2.9**	**96.4**
**15**	**5.3**	**98.3**	**122**	**2.1**	**99**	**1.7**	**118.3**
16	1.9	93.3	110.3	2.5	97.1	2.5	105.7
17	3.1	97.2	119.3	4.2	93	4.2	118.8
**18**	**4.3**	**104.7**	**107.7**	**2.9**	**98.3**	**2.9**	**110.3**
**19**	**5.8**	**108.5**	**106.7**	**2.5**	**106.2**	**2.1**	**98.9**
20	3.6	99.1	108.9	3.4	97.7	3.4	109.4
21	3.9	99.5	114.9	2.1	97.2	1.7	116.6
22	3.5	110.6	114	2.5	109	2.1	112.6
23	3.6	90.9	104.4	4.6	96	7.2	103.9
**24**	**6.1**	**108.4**	**99**	**4.2**	**107.4**	**4.2**	**100.7**
25	2.2	100	116.9	4	106.7	0	112.1
**26**	**4.5**	**114.7**	**107.9**	**4.2**	**113.9**	**3**	**111.5**
27	2.7	107.6	119.5	2.9	103.1	4.6	114.1
28	3.9	95.2	100.8	2.1	95.7	2.9	102.6
**29**	**9.2**	**101.2**	**106.4**	**10.5**	**99.3**	**10**	**108.7**
**30**	**7.8**	**95.5**	**117.9**	**8.4**	**92.3**	**8.4**	**112.3**
31	2.9	101.7	113.4	1.7	103.6	1.7	117.1
32	3.4	98	107.5	1.7	101.3	2.1	109.7
33	1.9	100.1	129.3	2.1	100.3	1.7	116.9
34	1.8	104.7	104.6	2.5	105.7	2.5	110.7
35	2.2	97	103.7	4.6	96.1	5	112.5
**36**	**4.2**	**92.4**	**118.4**	**6.7**	**94**	**7.1**	**125.4**
37	1.9	101.9	104.2	2.9	99.1	2.5	104.2
38	3.4	100.5	117	2.1	103.4	2.1	113.4
39	3.2	100.5	112.7	1.3	100.7	1.3	101.7
40	2.5	91.4	107.9	4.3	89.3	4.9	109.7
**41**	**4**	**97.6**	**101.1**	**3.4**	**95.8**	**3.4**	**103**
**42**	**5.4**	**104.2**	**97.8**	**2.1**	**101.8**	**2.1**	**105.3**

**Table 3:** Entire sample (42 subjects): linear regression results for OJ-r; only ANB and 21/SNA-SNP were found to be statistically significant. The systematic error calculated via Student's *t*-test for paired data yielded a significance level of 0.05; ns: nonsignificant.

	*B*	*P* level
ANB	0.64	0.000034
IMPA DX	−0.04	ns
11/SNA-SNP	−0.05	ns
21/SNA-SNP	0.16	0.004144
*R* ^2^ = 0.42954353; *R* ^2^ correct = 0.36787256		

**Table 4:** Entire sample (42 subjects): linear regression results for OJ-l; only ANB and 21/SNA-SNP were found to be statistically significant. The systematic error calculated via Student's *t*-test for paired data yielded a significance level of 0.05; ns: non significant (Student's *t*-test).

	*B*	*P* level
ANB	0.62	0.000144
11/SNA-SNP	−0.06	ns
IMPA SX	−0.08	ns
21/SNA-SNP	0.18	0.003007
*R* ^2^ = 0.40149186; *R* ^2^ correct = 0.33678828		

**Table 5:** Group 1 (25 subjects): linear regression results OJ-r; no independent variable was found to be statistically significant in the multivariate linear regression model. The systematic error calculated via Student's *t*-test for paired data yielded a significance level of 0.05; ns: non significant (Student's *t*-test).

	*B*	*P* level
ANB	0.30	ns
IMPA DX	−0.02	ns
11/SNA-SNP	−0.07	ns
21/SNA-SNP	0.12	ns
*R*² = 0.21405906; *R*² correct = 0.05687087		

**Table 6:** Group 1 (25 subjects): linear regression results for OJ-l; no independent variable was found to be statistically significant in the multivariate linear regression model. The systematic error calculated via Student's *t*-test for paired data yielded a significance level of 0.05; ns: non significant (Student's *t*-test).

	*B*	*P* level
ANB	0.65	ns
11/SNA-SNP	−0.06	ns
IMPA SX	−0.06	ns
21/SNA-SNP	0.11	ns
*R*² = 0.18725388; *R*² correct = 0.02470466		

**Table 7:** Group 2 (17 subjects): linear regression results for OJ-r: only the ANB variable was found to be statistically significant. The systematic error calculated via Student's *t*-test for paired data yielded a significance level of 0.05; ns: non significant (Student's *t*-test).

	*B*	*P* level
ANB	1.02	0.011533
IMPA DX	0.02	ns
11/SNA-SNP	0.03	ns
21/SNA-SNP	0.16	ns
*R*² = 0.54065844; *R*² correct = 0.38754459		

**Table 8:** Group 2 (17 subjects): linear regression results OJ-l. ANB and 21/SNA-SNP were found to be statistically significant. The systematic error calculated via Student's *t*-test for paired data yielded a significance level of 0.05; ns: non significant (Student's *t*-test).

	*B*	*P* level
ANB	1.04	0.006044
11/SNA-SNP	−0.04	ns
IMPA SX	−0.05	ns
21/SNA-SNP	0.22	0.03702
*R*² = 0.18725388; *R*² correct = 0.02470466		
